# Coronary Vasospastic Angina: A Review of the Pathogenesis, Diagnosis, and Management

**DOI:** 10.3390/life12081124

**Published:** 2022-07-27

**Authors:** Rajan Rehan, James Weaver, Andy Yong

**Affiliations:** 1 Concord Repatriation Hospital, Sydney, NSW 2137, Australia; rajan.rehan@sydney.edu.au; 2 Sydney Medical School, The University of Sydney, Sydney, NSW 2006, Australia; 3 Royal Prince Alfred Hospital, Sydney, NSW 2050, Australia; james.weaver@health.nsw.gov.au

**Keywords:** coronary artery vasospasm, vasospastic angina, endothelial dysfunction, intracoronary provocation testing

## Abstract

Vasospastic angina (VSA) is an under-appreciated cause of chest pain. It is characterised by transient vasoconstriction of the coronary arteries and plays a significant role in the pathogenesis of stable angina and acute coronary syndromes. Complex mechanistic pathways characterised by endothelial dysfunction and smooth muscle hypercontractility lead to a broad spectrum of clinical manifestations ranging from recurrent angina to fatal arrhythmias. Invasive provocation testing using intracoronary acetylcholine or ergonovine is considered the current gold standard for diagnosis, but there is a wide variation in protocols amongst different institutions. Conventional pharmacological therapy relies on calcium channel blockers and nitrates; however, refractory VSA has limited options. This review evaluates the pathophysiology, diagnostic challenges, and management strategies for VSA. We believe global efforts to standardise diagnostic and therapeutic guidelines will improve the outcomes for affected patients.

## 1. Introduction

Coronary vasospastic angina (VSA) is characterised by transient and reversible vasoconstriction of the coronary vasculature leading to myocardial ischemia. It was first described in a landmark report by Prinzmetal et al. in 1959 [1]. They reported 32 cases with a variant type of angina that occurred at rest with transient ST-segment elevation on electrocardiogram (ECG) [1]. Animal models demonstrated that this variant was a distinct functional disorder caused by intermittent hypertonic occlusion of the coronary arteries. Moving forward, Yasue et al. documented, by coronary arteriography, spasm of an epicardial coronary artery provoked by methacholine or exercise [2,3]. These early discoveries sparked interest amongst the wider cardiovascular community.

VSA is a heterogeneous phenomenon that can occur in patients with or without atherosclerosis and affects the epicardial coronary arteries or microvasculature. It is an under-appreciated cause of stable angina, acute coronary syndromes, malignant arrhythmias, and sudden cardiac death [4,5]. In recent years, interest in VSA has increased through research efforts by the global scientific community. Groups such as the Coronary Vasomotor Disorders International Study Group (COVADIS) and the Japanese Circulation Society (JCS) have focused on developing international recommendations for this condition. Nevertheless, the atypical clinical presentations, unpredictable symptoms, and varied responses to medical therapy render this condition difficult to manage. This review discusses the mechanistic pathways involved in the complex pathophysiology of VSA. In addition, we evaluate the current diagnostic approaches and management strategies available to clinicians.

## 2. Epidemiology

A survey from Japan revealed that up to 40% of patients with angina might have a component of VSA [6]; however, the exact prevalence is unknown. Although provocative testing during invasive coronary angiography has gained traction in recent years, it is still only performed at a few specialised centres. The majority of data on prevalence come from Asian populations, in particular Japan and Taiwan. These populations demonstrate a higher prevalence than Caucasians (24.3% and 19.3%, respectively, versus 7.5%) [7,8,9]. It is predominantly observed in adults aged 40–70 years [8,10]. Population studies have demonstrated a higher prevalence in males; however, these studies were confounded by increased tobacco consumption in this gender [11,12].

## 3. Pathophysiology

**Figure 1:**
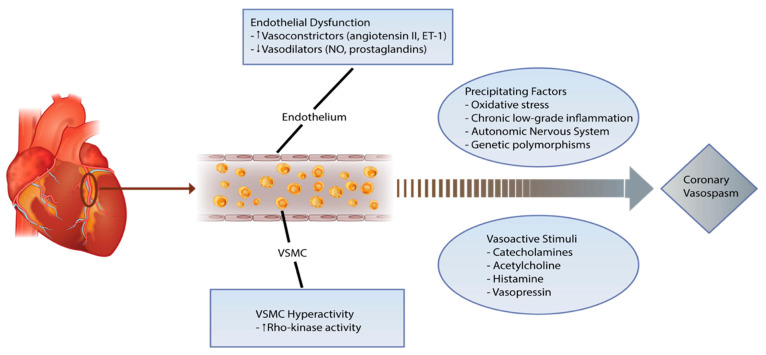
Complex pathophysiology of coronary vasospasm. Abbreviations: VSMC—vascular smooth muscle cells, ET-1—endothelin-1, NO—nitric oxide.

The pathophysiology of VSA is characterised by an impairment of vasomotion (physiological rhythmical contractions) in one or more coronary arteries [13]. In addition, vascular smooth muscle cell (VSMC) dysfunction, coupled with a predominance of vasoconstrictive metabolites, plays an adjunctive role [14,15]. A localised vasomotion abnormality may produce focal (segmental) spasm in a coronary artery, while a more diffuse abnormality may result in multivessel spasm [15,16]. The critical pathophysiological mechanisms implicated in VSA are summarised below (Figure 1).

*Vascular Smooth Muscle Hyper-Contractility:* The Rho-kinase enzyme, present in the VSMC, is a crucial regulator of hyper-contractility. Enhanced Rho-kinase activity results in excessive myosin light chain phosphorylation by inhibiting the myosin-binding subunit (MBS), leading to a state of hyper-contractility [14,15]. In addition, several additional pathways involving nitric oxide, phospholipase C, and KATP channels are also linked to VSMC hypercontractility [17,18,19]. These mechanistic pathways are primary contributors to VSA attacks.

*Endothelial Dysfunction:* A normally functioning endothelium in the coronary vasculature is responsible for producing nitric oxide (NO), a potent vasodilator [20]. It performs its function by suppressing vasoconstrictive metabolites such as angiotensin II and endothelium I [20]. Endothelial dysfunction results in a deficiency of endogenous NO, leading to a proliferation of vasoconstrictive metabolites. This mechanistic pathway clarifies why several endothelium-dependent vasodilators (e.g., acetylcholine, ergonovine, histamine, and serotonin) provoke vasoconstriction in patients with VSA [21]. Furthermore, it illustrates the heightened effect of endothelium-independent vasodilators (e.g., nitrates) in this setting [21].

*Chronic Inflammation:* The presence of several inflammatory biomarkers, including hsCRP, IL-6, and adhesion molecules, has emphasised the role of chronic low-grade inflammation in VSA [22]. This notion was reinforced by numerous animal and cellular models that revealed the same findings [23,24]. Unsurprisingly, persistent tobacco exposure is also associated with chronic inflammation [25]. Furthermore, the presence of coronary adventitial and perivascular adipose tissue inflammation was highlighted by Waters et al. in the tobacco-exposed population [26]. These findings demonstrate the significance of chronic inflammation in VSA.

*Autonomic Nervous System:* The overactivity of the sympathetic or parasympathetic nervous systems can contribute to VSA via the sensitisation of the VSMC and an increase in effector molecules such as acetylcholine and adrenaline. The nocturnal predominance of the condition, together with the capability of acetylcholine to induce VSA attacks, highlights the role of the parasympathetic system [27]. On the other hand, the increase in catecholamines and adrenergic activity following an episode of VSA represents the sympathetic system [28,29].

*Ethnicity:* Japanese and other Asian cohorts may show a higher prevalence and a more severe coronary artery involvement in VSA than White cohorts, which may be attributed to lifestyle and genetic differences [11,30]. Sueda et al. questioned these findings and highlighted fewer differences between Japanese and European cohorts [11]. A recent comparative international study found that among VSA patients, White patients had significantly lower rates of survival than did Japanese patients (76.6 vs. 86.7%) despite having similar patterns of coronary spasm on provocative testing [31].

*Genetic Polymorphisms:* Genetic mutations involving NO synthase, adrenergic receptors, serotoninergic receptors, angiotensin-converting enzyme (ACE), and paraoxonase I have been implicated in the pathogenesis of VSA [32,33,34,35]. In addition, several studies have demonstrated genetic polymorphisms that code for aldehyde dehydrogenase (ALDH), NADH/NADPH oxidase, and IL-6 [32,36,37]. Of note, circulating microRNA (miRs) contribute to the pathogenesis of endothelial nitric oxide synthase (eNOS) and may act as novel biomarkers for the diagnosis of VSA [38].

*Magnesium Deficiency:* Magnesium is an important naturally occurring antagonist of intracellular calcium. Up to 45% of VSA patients may have a magnesium deficiency, which may be an essential contributor to VSA in some patients [39,40].

*Atherosclerosis and Thrombosis:* Atherosclerosis and VSA are two discrete lesions that often coexist and share several risk factors [41]. The progression of either entity can lead to the aggravation of its counterpart. Coronary spasm can occur in both angiographically normal vasculature and atherosclerotic arteries [42,43], and it has a predisposition for branch points in coronary vessels and segments distal to stented lesions [44]. Given its complex pathophysiology, most believe that lesions in coronary spasm are dissimilar to traditional lipid-laden atherosclerosis [42,43]. Another point of interest is the relationship between VSA and thrombosis. Elevated levels of plasminogen activator inhibitor 1 and fibrinopeptide A have been demonstrated following VSA attacks [45]. Furthermore, platelets are activated after VSA attacks but not after stable angina [46]. These findings suggest that prolonged attacks of spasm can trigger coronary thrombosis.

*Acute Coronary Syndrome:* Coronary artery spasm has a crucial etiologic role in the pathogenesis of ACS. The CASPER study demonstrated that one-third of patients who presented with NSTE-ACS and no obstructive lesion had evidence of coronary spasm [47]. The mechanisms involved include prolonged coronary spasm leading to vessel occlusion and subsequent myocardial ischemia, coronary plaque progression and rupture of vulnerable plaque, and acute thrombus formation [48,49,50,51]. Kobayashi et al. used intravascular optical coherence tomography to examine the culprit lesion of patients with coronary spasm and ACS, which demonstrated a reduction in the luminal area with vascular contraction and thrombus formation without atherosclerotic plaque disruption [52].

## 4. Clinical Spectrum

The clinical spectrum of VSA is variable but essentially includes a transient, non-exertional, nitrate-responsive angina that tends to occur at night or in the early morning. A study at the Cleveland Clinic of 59 patients with VSA demonstrated that angina at rest (93%) was the predominant symptom [53]. This was reiterated by Bertrand et al., who performed invasive provocative testing in 1089 consecutive patients in which coronary artery spasm was present in 38% of patients with rest angina only, 14% with rest and exertional angina, 4% with only exertional angina, and 1% with atypical chest pain [7]. Such chest discomfort can be accompanied by cold sweats, nausea, vomiting, and syncope, particularly when it is severe. Symptoms are typically relieved by short-acting nitrates and calcium channel blockers (CCBs), although a small fraction of patients can be refractory to such therapy.

Interestingly, the natural history of VSA remains a topic of discussion. Several studies have recognised the spontaneous remission of symptoms [53,54,55]. As previously mentioned, Bott-Silverman et al. indicated that 39% of patients from their study cohort were asymptomatic at six months and 15% at two years. Nevertheless, 5 of the 14 patients with long-term remission eventually had a reoccurrence of symptoms [53]. Given the presence of silent myocardial ischemia in many such patients, a lack of anginal symptoms may not always indicate a benign prognosis. Furthermore, such patients can suffer prolonged bouts of vasospasm, leading to the progression of atherosclerosis, thrombus formation, and eventually myocardial infarction [42,43].

The exact mechanism of the circadian variation in symptoms is yet to be clarified, although the predominantly nocturnal activity of the autonomic nervous system is thought to play a significant role [56,57]. In addition, circadian variations in numerous hormones, including catecholamines, cortisol, vasopressin, melatonin, growth hormone, and insulin, as well as variations in inflammatory cytokines such as TNF- and IL-1, play a presumed role [14,58]. Although uncommon, Kounis syndrome, also known as ‘allergic angina’, is an atypical form of VSA worth mentioning. It is characterised by the simultaneous occurrence of coronary spasm and a hypersensitivity reaction [59,60]. The unique mechanistic pathway involves the release of inflammatory cytokines through mast cell activation in the context of specific environmental exposures or drugs. Given its unique mechanism, the prompt recognition of this condition is important because it requires a different treatment approach [61,62,63].

*ECG Changes:* The ECG can be unremarkable in mild or early VSA. In contrast, severe spasm of a major epicardial artery can produce transient ischemic changes in leads that reflect the subtended myocardium. Common transient ischemic ECG changes in VSA include peaked and symmetrical T waves, an ST elevation ≥ 0.1 mV, an ST depression ≥ 0.1 mV, the emergence of negative U waves, an absent S wave, and prominent R waves [64,65]. In approximately half of patients with focal proximal coronary spasm, the presence of peaked and symmetrical T waves is evident [64]. If spasm persists, progressive ST-segment elevation with reciprocal ST-segment depression may occur [14]. Such ECG changes indicate a total or subtotal occlusion, whilst primary ST depression represents sub-endocardial ischemia. As highlighted by Bott-Silverman et al., most patients had a normal resting ECG but demonstrated significant ST elevation (64%) or ST depression (17%) during spontaneous anginal attacks [53]. ECG changes may be highly variable in the same patient during spontaneous and provoked episodes. Various arrhythmias may be observed, such as premature ventricular complexes, ventricular tachycardia, ventricular fibrillation, supraventricular arrhythmias, atrioventricular blocks, asystole, and supraventricular arrhythmias [64]. Interestingly, Suzuki et al. demonstrated an increased baseline QTc dispersion in patients with VSA, which indicated a higher susceptibility to ventricular arrhythmias [66].

*Comorbidities and Precipitating Factors:* Overall, the comorbidities of VSA have received little attention in the literature. A recent analysis described the comorbidity profiles of 96,901 VSA hospitalisations; the results revealed a high burden of hypertension (60.3%), dyslipidaemia (37.8%), chronic lung disease (20.7%), and diabetes (18.2%) [67]. Moreover, the presence of heart failure; chronic kidney, lung, or liver disease; and a previous history of MI significantly contributed to mortality [67]. Besides these potential relationships, the association of VSA with migraine and Raynaud’s phenomenon have encouraged speculations that VSA may represent the coronary component of a systemic vasomotor syndrome [68]. The presence of several risk factors increases the presence of VSA attacks, which can ultimately lead to a self-perpetuating cycle. Notably, several triggers for VSA episodes have been described in the literature (Table 1). These can trigger an episode of VSA by provoking an imbalance between coronary vasoconstrictors and vasodilators.

## 5. Diagnostic Approach

The diagnosis of VSA is based on the following three considerations: (i) the typical clinical presentation of VSA, (ii) the evidence of transient ischemia on ECG during the angina episode, and (iii) the demonstration of a spontaneous or provoked coronary vasospasm [69,70]. The question of when to undergo invasive or non-invasive testing to diagnose VSA has been addressed extensively by the JCS and COVADIS guidelines (Table 2) [10,69]. The JCS guidelines recommend invasive testing in suspected VSA patients with borderline ECG changes and those diagnosed based on ECG who are partial/non-responders to medical treatment [10]. In addition to these indications, the COVADIS guidelines have expanded provocative testing in other conditions, including ischemia with non-obstructive coronary arteries (INOCA), myocardial infarction with non-obstructive coronary arteries (MINOCA), unexplained cardiac arrest, unexplained syncope, and residual angina after successful percutaneous intervention (PCI) [69]. Patients without symptoms of VSA should not receive provocative testing according to both guidelines. Additional contraindications to provocative testing include left main narrowing (>50%), triple-vessel disease, two-vessel disease with total occlusion, heart failure (New York Heart Association Class III or IV), renal failure (creatinine > 2.0 mg/dL), and the presence of spontaneous spasm [10,69,71]. In addition, the presence of severely asthmatic bronchioles is an independent contraindication for ACh.

Provocative testing for VSA can be based on a variety of stimuli, both physiological (hyperventilation or cold exposure) or pharmacological (ergonovine, acetylcholine, neuropeptide Y, or dopamine), which can be used independently or in combination [72,73,74,75,76]. The chosen pharmacological route can also differ, e.g., intravenous, intraarterial, or intracoronary. To clinch the diagnosis, the clinician may use a non-invasive assessment, such as ECG or echocardiography, or an invasive method, such as coronary angiography. These choices of stimuli, routes, and assessment methods are often coupled with the preferences of clinicians, based on diagnostic yield, safety, availability, and individual experience [77].

*Invasive provocative testing:* Invasive pharmacological provocation testing is considered the current gold standard. Recent literature highlights the superiority of intracoronary acetylcholine and ergonovine in diagnosing coronary vasospasm. Nevertheless, the lack of high-grade evidence has led to diverse protocols. Overall, recommendations by the JCS and the COVADIS working group have gained the most popularity, though the need for a uniform, evidence-based protocol is critical [10,69]. All provocative pharmacological tests should be performed in the morning, and patients are requested to discontinue medications (including CCB, B-blockers, nitrates, ACE-I/ARB, and mineralocorticoid inhibitors) as well as caffeine, ideally for 48 h prior to the procedure [77,78,79].

Acetylcholine (ACh) is a vasoactive substance that provokes vasospasm via cholinergic receptors on vascular smooth muscle cells. A healthy endothelium responds to ACh through vasodilatation via nitric oxide (NO) release, whilst a dysfunctional endothelium cannot release enough NO to counteract the stimulated muscarinic receptors leading to vasoconstriction [20]. Such mechanistic properties have made ACh provocation testing a popular choice. Doses differ for left (LCA) and right (RCA) coronary arteries but are consistently higher for the left system [10,69,77,78]. The maximum dosage for the LCA is 200 µg, and for the RCA it is 80 µg. Most protocols have an incremental dosing regimen and initiate testing in the LCA. The administration time of ACh during provocation testing varies between a 3-min infusion and a 20-s manual injection [10,80,81,82,83]. Despite a marginal increase in overall procedural time, the insertion of a temporary pacemaker during provocative testing is encouraged by most international groups if testing the RCA [71,82].

Another popular agent is ergonovine, a vasoactive substance that mainly acts via the serotonergic receptors in vascular smooth cells leading to vasoconstriction [84,85,86]. Initially, ergonovine provocative testing involved intravenous administration. However, safety concerns highlighted by a report of three deaths secondary to ventricular arrhythmias led to the emergence of intracoronary administration [87,88]. Like with ACh, provocation testing doses of ergonovine vary between the LCA and RCA, with maximum doses of 80 µg and 60 µg, respectively [89,90,91]. Most protocols follow an incremental dosing regimen starting at a minimum dose of 10–20 µg for either coronary artery [89,90,91]. The administration time has been significantly reduced over the last decade from a 240-s [92] to a 60-s infusion [89,90,91].

The preference for acetylcholine or ergonovine is still being debated, although acetylcholine is more widely used internationally. Acetylcholine has the advantage of rapid onset and reversibility, but the use of ergonovine appears to have a lower incidence of arrhythmias [82,93]. Selection may also be influenced by gender (acetylcholine may be favoured in females) and age (ergonovine may be favoured in younger patients) [93]. In addition, clinician expertise and experience play a crucial role.

*Non-Invasive:* Given their safety, non-invasive, provocative testing approaches have a role in the diagnostic algorithm for VSA. Due to low accuracy, they are not used as definitive tests but may be considered helpful screening modalities. The JCS 2013 guideline recommends hyperventilation and exercise as valid methods of non-pharmacological provocative testing, along with ECG monitoring [10]. The guideline suggests a target respiratory rate of 25 respirations per minute (for up to 6 min) with discontinuation of the test in the presence of angina or significant ECG changes. The COVADIS guidelines also recommend hyperventilation as a valid option but prefer cold pressor testing instead of exercise [69]. This sympathetic reflexogenic stimulus can provoke coronary spasm, though pharmacological agents have primarily replaced it. Waters et al. reported better sensitivity with IV ergonovine than with non-pharmacological techniques in the early 1980s [72]. Subsequently, Song et al. demonstrated the safety of IV ergonovine in 1372 patients [94]. Recently, Om et al. assessed 14,012 patients with ergonovine echocardiography. They found that 15.3% of patients had coronary spasm demonstrated by ECG changes or reversible regional wall motion abnormalities on echocardiography [95]. These results highlight the emergence of non-invasive testing; however, further data on sensitivity and specificity are required to determine its role in clinical practice.

*Intracoronary Imaging:* Intracoronary imaging using intravascular ultrasound (IVUS) and optical coherence tomography (OCT) has an emerging but limited role in evaluating VSA. IVUS can identify plaque composition and intimal hyperplasia at the site of focal coronary artery spasm in the absence of significant angiographic disease [96,97,98]. In conjunction, OCT can accurately delineate structural changes in spastic coronary arteries [99]. The characteristic abnormalities in such patients include the presence of an intimal bump and thickened media during the spasm; these abnormalities resolve after nitro-glycerine administration [99,100].

## 6. Management

**Figure 2:**
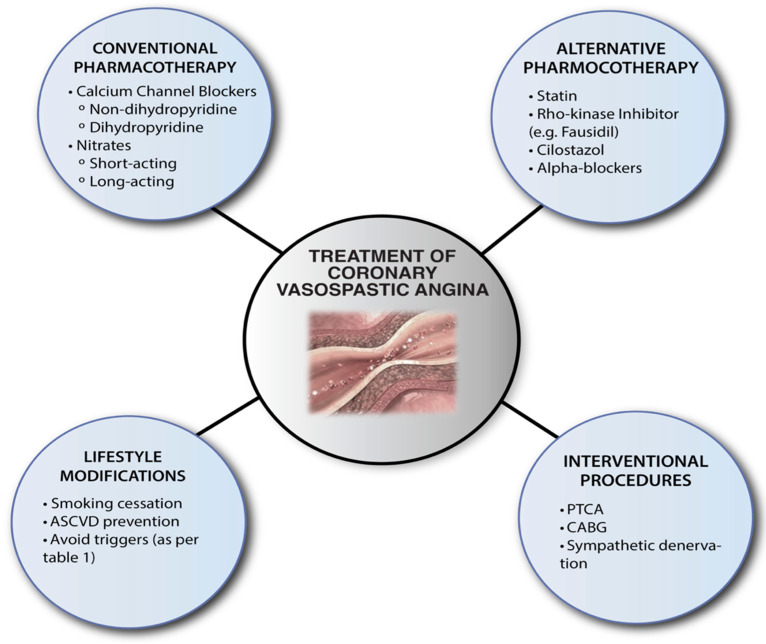
Summary of current therapeutic strategies for coronary vasospastic angina. ASCVD = atherosclerotic cardiovascular disease, PTCA = percutaneous transluminal coronary angioplasty, CABG = coronary artery bypass grafting.

The optimal management of VSA includes lifestyle modifications, conventional pharmacotherapy, alternative pharmacotherapy, and cardiac interventions for selected clinical subsets (Figure 2).

*Lifestyle Modifications:* Given the presence of endothelial dysfunction in such patients, the avoidance of precipitating factors that impair endothelial function or increase oxidative stress (Table 1) are seen as pillars in management. Smoking cessation is critical in patients with CAS as chronic tobacco exposure is a leading risk factor [25,101]. Alcohol consumption may induce attacks several hours after consumption, especially in patients with the aldehyde dehydrogenase (ALDH2) polymorphism [102]. Emotional stress, hyperventilation, and exposure to extreme cold may also precipitate attacks [103,104,105]. Magnesium deficiency should be promptly addressed with supplementation, given its role as an endogenous calcium antagonist [39,40]. Furthermore, pharmacological triggers (Table 1) should be avoided.

*Conventional Pharmacotherapy:* CCBs and nitrates are among the most effective evidence-based guideline-recommended treatment options for VSA; thus, they form the mainstay of medical treatment [106,107]. CCBs remain the drugs of choice, while nitrates are the preferred add-on drugs for partially or non-responsive patients; however, they can also be used alone. CCBs inhibit calcium inflow into the VSMCs and stimulate NO production, leading to vasodilation [108]. Given the circadian variability of VSA, these drugs should be administrated at night. Doses should be steadily increased to optimise angina status and avoid adverse effects. For severe symptoms, the combination of dihydropyridine and non-dihydropyridine CCBs should be considered [109]. Benidipine, a long-acting dihydropyridine CCB, has demonstrated a prognostic benefit in patients with VSA [110]. Alternatively, non-dihydropyridine CCBs have displayed superior efficacy in suppressing angina attacks [110]. In patients with substantial prognostic risk factors (e.g., multi-vessel spasm, elevated hsCRP, and cardiac arrest), CCBs should be continued regardless of symptoms given the risk of silent myocardial ischemia, which can result in ventricular arrhythmias and sudden cardiac death [14]. Nitrates lead to coronary vasodilatation through in vivo conversion to NO. These agents replace endogenous endothelial NO, leading to a reduction in angina episodes. Short-acting nitrates are very effective for prompt relief, whilst long-acting nitrates act preventatively. Notably, chronic organic nitrate use can result in unwanted side effects. Regular consumption can lead to a rise in reactive oxygen species (ROSs), which degrade NO and further increase the frequency of VSA attacks [111,112]. In contrast to traditional effort angina, beta-blockers are thought to aggravate symptoms through an increased vasoconstrictive response [113]. Only nebivolol, given its high beta-1 affinity, can foster vasodilation through NO production [114].

*Alternative Pharmacotherapy:* Various alternative vasodilators and antioxidants have been explored in patients refractory to standard medical therapy. Statins are thought to suppress anginal attacks by improving overall endothelial function through the augmentation of endothelial NO activity combined with the suppression of the RhoA/ROCK pathway [115,116,117]. These off-target effects can improve symptoms and overall prognosis. Fasudil is a specific Rho-kinase inhibitor highly effective in preventing ACh-induced coronary spasm [118]. The Rho-kinase pathway is involved in endothelial dysfunction, vasospasm, and inflammatory cell accumulation. Fasudil can target these pathogenic mechanisms, leading to a reduction in myocardial ischemia and symptoms of angina [119,120]. It is effective for patients with epicardial coronary spasm and approximately two-thirds of patients with microvascular spasm [121]. Cilostazol is a phosphodiesterase (PDE) type III inhibitor used for intermittent claudication, post-CVA prevention, and coronary stent restenosis [122]. The inhibition of PDE increases intracellular cyclic adenosine monophosphate, a substance with anti-inflammatory and vasodilatory effects. Mohri et al. highlighted the promising role of cilostazol in VSA patients through an improvement in CFR and coronary volumetric flow [123]. Furthermore, a placebo-controlled randomised trial for treatment-refractory patients demonstrated a reduction in angina frequency and intensity [124]. Other agents to consider in treatment-refractory VSA are nicorandil (a nitrate and K-channel activator) [125] and pioglitazone (a peroxisome-proliferator-activated receptor agonist) [126]. In addition, clinicians may consider the concomitant administration of magnesium and antioxidants (e.g., Vitamin C and E) for an additional benefit [127,128].

*Interventional Treatment:* Several interventions have been attempted in complicated non-responsive VSA, though available evidence supporting their use remains limited. Coronary artery bypass grafting (CABG) and percutaneous coronary intervention (PCI) can be considered in patients with significant epicardial stenosis. The success of such procedures is favoured in patients with focal, non-diffuse atherosclerotic stenosis [129,130]. Of note, particularly in PCI, the reoccurrence of CAD distal to the angioplasty site is a known phenomenon [129,131]. Sympathetic denervation remains an option for severe refractory VSA, although only a few cases with favourable outcomes have been described [132]. The implantation of a cardiac defibrillator (ICD) is reserved for secondary prevention in CAS patients with aborted sudden cardiac death or ventricular arrhythmias on appropriate medical therapy [133,134,135]. There is no concrete evidence for ICD implantation in primary prevention.

## 7. Prognosis

The prognosis of VSA is generally favourable compared to epicardial atherosclerotic coronary stenosis [43,136]. Waters et al. demonstrated a survival rate of 95%, 90%, and 87% at 1, 2, and 3 years, respectively [137], which mirrored studies from Japan and Korea [91,136,138]. Nevertheless, the contemporary literature suggests a high morbidity, with half of adequately treated patients experiencing reoccurring angina [110,139]. Furthermore, coronary artery vasospasm may lead to myocardial infarction, fatal arrhythmias, or sudden cardiac death, highlighting the importance of appropriate diagnosis [140,141,142]. Of note, the prognosis of patients with VSA and aborted sudden cardiac death (ASCD) is far worse than that of their counterparts [135]. Advanced age, multi-vessel spasm, elevated hsCRP, and out-of-hospital cardiac arrest are other significant predictors of mortality [67,143]. In recent years, the Japanese Coronary Spasm Association has developed a risk stratification score to prognosticate patients with VSA [144]. Most studies agree that treatment with CCBs and abstinence from smoking are crucial prognostic markers.

## 8. Conclusions

Coronary vasospastic angina is an under-appreciated cause of chest pain. It is a significant but often overlooked contributor to obstructive and non-obstructive coronary syndromes, ventricular arrhythmias, and sudden death. Endothelial dysfunction, vascular smooth muscle cell hypercontractility, and the predominance of vasoconstrictive metabolites characterise its complex pathogenesis. Invasive pharmacological provocation testing remains the gold standard for diagnosis, though various protocols exist. Lifestyle modifications and conventional pharmacotherapy outline current management, although the treatment of refractory cases remains an ongoing challenge. Further research and the development of a universally accepted diagnostic and therapeutic pathway will improve the quality of care received by this cohort of patients.

## Figures and Tables

**Table 1:** Precipitating factors for vasospastic angina.

Physiological	Pharmacological
● Stress—mental and physical	● Catecholamines
● Early-morning exertion	● Cholinergic agents
● Cold exposure	● Serotonergic agents
● Hyperventilation	● Beta-blockers
● Valsalva manoeuvre	● CNS stimulants
● Magnesium deficiency	● General anaesthesia
● Activated platelets	● Chemotherapeutic agents
● Procedural manipulation of coronary arteries	● Tobacco and alcohol

**Table 2:** Indications for coronary provocation testing.

COVADIS Group	Japanese Circulation Society
Class I - History of suspected VSA without documented spontaneous episodes, especially in cases of: - Acute coronary syndrome presentation in the absence of a culprit lesion - Unexplained resuscitated cardiac arrest - Unexplained syncope with antecedent chest pain - Recurrent rest angina following angiographically successful PCI	Class I - VSA is suspected based on symptoms, but in those who have not been diagnosed with coronary spasm by non-invasive evaluation
Class IIa - Invasive testing for non-invasively diagnosed patients unresponsive to drug therapy	Class IIa - Patients who have been diagnosed with coronary spasm by non-invasive evaluation, and medical treatment is ineffective or insufficiently effective
Class IIb - Documented spontaneous episode of variant angina - Invasive testing for non-invasively diagnosed patients responsive to drug therapy	Class IIb - Patients who have been diagnosed with coronary spasm by non-invasive evaluation, and medical treatment has been proven to be effective
Class III - Emergent acute coronary syndrome - Severe fixed multivessel CAD including LMT artery stenosis - Severe myocardial dysfunction (Class IIb if symptoms are suggestive of vasospasm) - Patients without any symptoms suggestive of VSA	Class III - Emergent coronary angiography in patients with acute coronary syndrome - High risk of suffering life-threatening complications induced by coronary spasm (LMT/MVD including obstructive lesions) - Severe cardiac dysfunction or CHF (Class IIb if it may be a consequence of vasospasm) - Patients without symptoms suggestive of VSA

## Data Availability

Not applicable.
